# Planktonic Aggregates as Hotspots for Heterotrophic Diazotrophy: The Plot Thickens

**DOI:** 10.3389/fmicb.2022.875050

**Published:** 2022-04-06

**Authors:** Lasse Riemann, Eyal Rahav, Uta Passow, Hans-Peter Grossart, Dirk de Beer, Isabell Klawonn, Meri Eichner, Mar Benavides, Edo Bar-Zeev

**Affiliations:** ^1^Marine Biology Section, University of Copenhagen, Helsingør, Denmark; ^2^Israel Oceanographic and Limnological Research, Haifa, Israel; ^3^Ocean Science Centre, Memorial University of Newfoundland, St. John’s, NL, Canada; ^4^Institute for Biochemistry and Biology, Potsdam University, Potsdam, Germany; ^5^Department of Plankton and Microbial Ecology, Leibniz-Institute of Freshwater Ecology and Inland Fisheries (IGB), Stechlin, Germany; ^6^Max Planck Institute for Marine Microbiology, Bremen, Germany; ^7^Department of Biological Oceanography, Leibniz Institute for Baltic Sea Research, Rostock, Germany; ^8^Institute of Microbiology CAS, Centre ALGATECH, Třeboň, Czechia; ^9^Aix Marseille Univ, Université de Toulon, CNRS, IRD, MIO, Marseille, France; ^10^Turing Center for Living Systems, Aix-Marseille University, Marseille, France; ^11^The Jacob Blaustein Institutes for Desert Research, Zuckerberg Institute for Water Research (ZIWR), Ben-Gurion University of the Negev, Be’er Sheva, Israel

**Keywords:** aggregates, nitrogen fixation, heterotrophic bacteria, marine, aquatic, NCDs

## Abstract

Biological dinitrogen (N_2_) fixation is performed solely by specialized bacteria and archaea termed diazotrophs, introducing new reactive nitrogen into aquatic environments. Conventionally, phototrophic cyanobacteria are considered the major diazotrophs in aquatic environments. However, accumulating evidence indicates that diverse non-cyanobacterial diazotrophs (NCDs) inhabit a wide range of aquatic ecosystems, including temperate and polar latitudes, coastal environments and the deep ocean. NCDs are thus suspected to impact global nitrogen cycling decisively, yet their ecological and quantitative importance remain unknown. Here we review recent molecular and biogeochemical evidence demonstrating that pelagic NCDs inhabit and thrive especially on aggregates in diverse aquatic ecosystems. Aggregates are characterized by reduced-oxygen microzones, high C:N ratio (above Redfield) and high availability of labile carbon as compared to the ambient water. We argue that planktonic aggregates are important loci for energetically-expensive N_2_ fixation by NCDs and propose a conceptual framework for aggregate-associated N_2_ fixation. Future studies on aggregate-associated diazotrophy, using novel methodological approaches, are encouraged to address the ecological relevance of NCDs for nitrogen cycling in aquatic environments.

## Introduction

Biological dinitrogen (N_2_) fixation, the conversion of dissolved N_2_ into ammonia, can represent a critical import of reactive nitrogen to the pelagic environment (Karl et al., 2002). This process is carried out by specialized prokaryotic microorganisms termed diazotrophs (Zehr and Turner, 2001). Aquatic studies have traditionally focused on photoautotrophic cyanobacterial diazotrophs inhabiting oligotrophic and sunlit environments where energy is made available *via* photosynthetic carbon fixation (Zehr, 2011). However, during the last decade it has become evident that non-cyanobacterial diazotrophs (NCDs; see Box 1) have an almost ubiquitous distribution in pelagic environments (Farnelid et al., 2011; Langlois et al., 2015; Geisler et al., 2020; Hallstrøm et al., 2021; Messer et al., 2021). For instance, the presence and/or expression of the nitrogenase gene (*nifH*) by NCDs has been reported from low latitude open oceans (Halm et al., 2011; Moisander et al., 2014; Langlois et al., 2015) to environments previously not regarded as suitable for N_2_ fixation such as eutrophic rivers, estuaries and coastal waters (Mulholland et al., 2012; Bentzon-Tilia et al., 2015; Geisler et al., 2020; Hallstrøm et al., 2021), the aphotic deep sea (Rahav et al., 2013; Benavides et al., 2015), and nutrient-rich arctic waters (Harding et al., 2018). A recent study suggested that some *nifH* genes are not functional (Mise et al., 2021). Yet, these genes relate to obligate anaerobic bacteria and their prevalence in the marine pelagic environment is likely minor. The activity of NCDs has been indirectly inferred by experimental manipulations that inhibit photoautotrophic activity (Rahav et al., 2016; Benavides et al., 2018b; Geisler et al., 2019, 2020) and from environments putatively void of cyanobacteria such as aphotic waters (Hamersley et al., 2011; Rahav et al., 2013; Benavides et al., 2015). Still, these methods cannot measure NCD-specific N_2_ fixation rates unambiguously. Thus, despite of the widespread distribution and activity of NCDs, their contribution to aquatic nitrogen cycling remains elusive (see reviews: Riemann et al., 2010; Bombar et al., 2016; Moisander et al., 2017; Benavides et al., 2018a; Marcarelli et al., 2022).

BOX 1 **Key term definitions.**

**Non-cyanobacterial diazotrophs (NCDs):** From a phylogenetic, rather than a metabolic, point of view, diazotrophs can be divided into cyanobacteria and non-cyanobacteria. The term NCDs has therefore been widely used in the literature. NCDs are a diverse group of prokaryotes with potentially diverse metabolic pathways (see definition below). In the context of bacterial growth and N_2_ fixation associated with aggregates, we consider degradation and uptake of organic matter (heterotrophy) the prevailing functionality. However, the reader should be aware that other metabolic strategies such as mixotrophy, photoheterotrophy or chemolithoautotrophy may also be present in NCDs.

**Heterotrophic diazotrophs:** Archaeal and bacterial N_2_-fixing microorganisms that require organic matter from external sources.

**Metabolism:** The combination of energy sources (light, chemical, and organic matter), electron flow and carbon (CO_2_ or organic matter) used by a microorganism to catalyze catabolic or anabolic processes.

**Aggregates:** Particles comprising live, dead and/or dormant cells, detritus and minerals that are held together by organic scaffolds. These particles are formed by the aggregation of organic material suspended in seawater. Aggregates are often rich in labile carbon and nutrients, and are therefore hotspots of microbial activity.

The marine water column is generally well oxygenated (except for oxygen minimum zones) and poor in labile organic matter (Arrieta et al., 2015; Santinelli, 2015), whereas the aphotic zone is rich in reactive nitrogen (e.g., Cavender-Bares et al., 2001). Therefore, the wide distribution of NCDs in these habitats with apparent unfavorable conditions for diazotrophy represents a lingering enigma. In this mini-review, we compile recent reports related to NCDs and focus on those associated with aggregates. We argue that the plot thickens [*sensu* (Azam, 1998)], and that compelling evidence supports the idea of planktonic aggregates as important microenvironments suitable for NCD N_2_ fixation. We emphasize the need for direct *in situ* measurements of aggregate-associated, NCD-specific N_2_ fixation, and provide guidelines for how these can be obtained in future studies. We note that this review paper focuses on marine and estuarine environments, as most data are available from such environments, but acknowledge that NCDs are also found in freshwater ecosystems (Coyne et al., 2020; Fernandez et al., 2020; Geisler et al., 2020).

## The Plot Thickens: Previous and New Insights on Aggregate-Associated N_2_ Fixation

Aggregates are ubiquitous throughout marine and freshwater environments (Alldredge and Gotschalk, 1988; Waite et al., 2000). They are formed by the coagulation of live and dead plankton material (Smith et al., 1992; Grossart and Ploug, 2000; Piontek et al., 2009; Daly et al., 2016). The elevated micronutrient and macronutrient concentration relative to the surrounding waters fosters colonization by dense communities of prokaryotes (del Giorgio and Cole 1998; Simon et al., 2002; Bar-Zeev and Rahav 2015), making aggregates ‘hot spots’ of intense microbial activity (Azam and Long, 2001). More than three decades ago, Hans Paerl and co-workers (Paerl, 1985; Paerl and Prufert, 1987) suggested that NCD N_2_ fixation may take place in low oxygen microzones within aggregates. This idea was reiterated in several later studies (Riemann et al., 2010; Sohm et al., 2011; Bombar et al., 2016), but has been substantiated only most recently (see below).

In the past, and especially during the last decade, evidence has accumulated for the association of NCDs with aquatic organisms and aggregates. NCDs have been isolated from copepods (Proctor, 1997) and *nifH* genes have been amplified and sequenced from copepods and euphausiids (Braun et al., 1999; Scavotto et al., 2015), and dinoflagellates (Farnelid et al., 2010). Moreover, individual and bulk aggregates collected with sediment traps deployed at 150 m depth in the open ocean contained *nifH* gene sequences of diverse NCDs (Farnelid et al., 2018). The prevalence of NCDs on aggregates has also been reported using metagenomics sequencing. In the Tara oceans dataset, representing 197 globally distributed pelagic oceanic metagenomes, the putative heterotrophic Planctomyces and Proteobacteria accounted for ~25% of the *nifH* reads obtained from the 180 to 2,000 μm size-fraction (Karlusich et al., 2021). Moreover, metagenome assembled genomes representing NCDs occurred in the 5–2,000 μm planktonic size-fractions (Delmont et al., 2021). Finally, one of the most widely distributed NCDs, Gamma-A, showed a ubiquitous presence in *nifH* genes across the North Atlantic Ocean quantified by qPCR in the >3 μm fraction (Benavides et al., 2016). This Gamma-A was also found in metatranscriptomes from the 3 to 2,000 μm size-fraction in the Tara oceans dataset, suggesting a filamentous or aggregate-attached lifestyle for this putative heterotrophic bacterium (Cornejo-Castillo and Zehr, 2020). Hence, both PCR-dependent and -independent approaches suggest the presence and/or activity of NCDs on aggregates.

**Figure 1:**
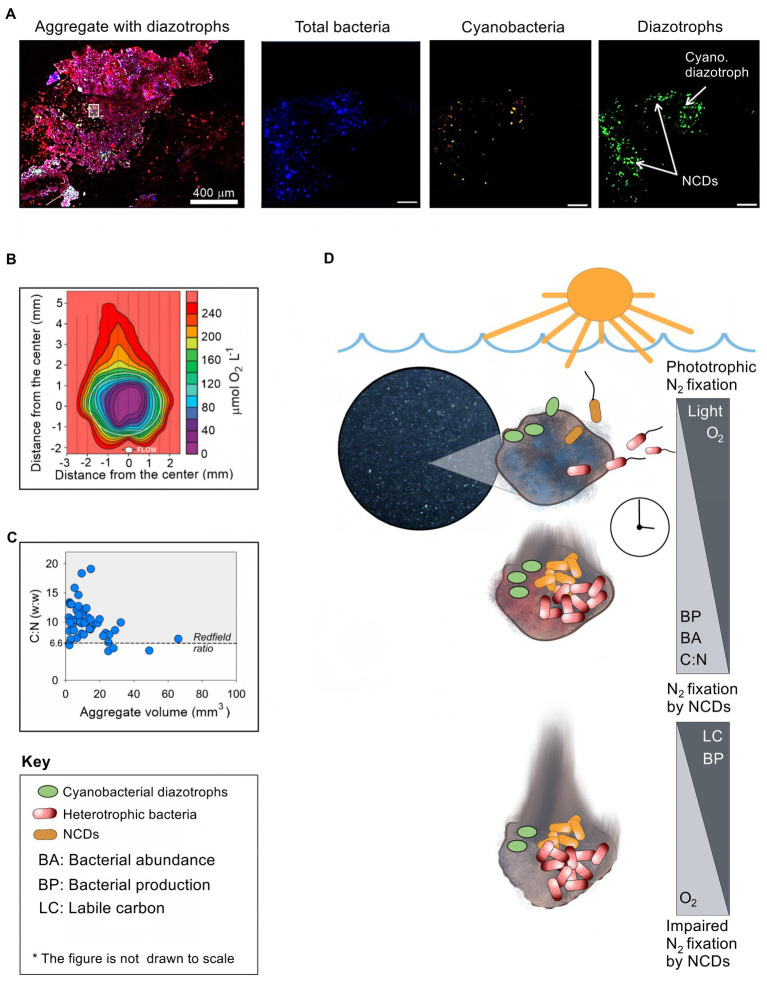
The association of NCDs with planktonic aggregates. **(A)** Enlarged confocal images of diazotrophs associated with aggregates after staining (red) the polysaccharide matrix by concanavalin A (Geisler et al., 2022). Enlarged images within the aggregate were captured to differentiate between three distinct channels (from left to right): total bacteria (stained by DAPI, blue); total cyanobacteria by autofluorescence of the phycoerythrin pigment (orange/white), and diazotrophs by immunolabeling nitrogenase enzyme. The white square on the aggregate shows the magnified location (scale bar of 10 μm). **(B)** Micro-sensor image showing the oxygen levels within a large (> 0.5 mm) planktonic aggregate (Klawonn et al., 2015). **(C)** Carbon to nitrogen ratio relative to aggregate size (Alldredge and Silver, 1988; Grossart and Ploug, 2001). **(D)** Conceptual figure illustrating time-course changes in conditions on an aggregate as it sinks in the water column, of key relevance for N_2_ fixation by associated NCDs. See text for explanation.

Experimental data also suggest aggregate-associated N_2_ fixation by NCDs. In an early study from the Chesapeake Bay, United States, experiments by Guerinot and Colwell (1985) suggested that isolated strains of NCDs could fix N_2_ in the presence of plankton and particulate matter. In an experiment with aggregates from the Southern Indian Ocean, *nifH* genes related to Deltaproteobacteria were enriched in metatranscriptomes from experimental incubations with aggregates relative to controls without aggregates (Debeljak et al., 2021). Similarly, N_2_ fixation was stimulated in seawater from a Danish nutrient rich estuary and the Mediterranean Sea by amendment with natural aggregates (Pedersen et al., 2018) or a transparent exopolymer aggregate analog (gum-xanthan; Rahav et al., 2016), respectively. Hence, the presence of aggregates appears to stimulate N_2_ fixation by NCDs. Finally, presence of NCDs was recently documented on aggregates by immunolabeling of the nitrogenase enzyme while at the same time superimposing the aggregate matrix, total bacteria and cyanobacteria (Figure 1A). Collectively, the above-mentioned findings suggest that NCDs benefit from colonizing aggregates. Yet, our mechanistic understanding of how aggregates support N_2_ fixation by NCDs is still rather limited.

## How Can Aggregates Support Heterotrophic Diazotrophy?

Theoretical considerations as well as experimental and field observations indicate that aggregates provide several conditions, which at least ephemerally, can support N_2_ fixation by NCDs: (1) *Low oxygen conditions:* Nitrogenase, a central enzyme for N_2_ fixation, is irreversibly damaged by molecular oxygen (Goldberg et al., 1987); however, aerobic respiration by bacteria that colonize the aggregate combined with slow diffusion rates (depending on the size and density of the particle) leads to local reduction in oxygen concentrations (Alldredge and Cohen, 1987; Paerl and Prufert, 1987; Ploug et al., 1997; Simon et al., 2002; Klawonn et al., 2015). The low oxygen levels in some parts of the aggregate vary from 50% to 90% air-saturation to anaerobic conditions on some occasions inside compact and large (few mm) aggregates (Ploug et al., 1997; Ploug, 2001; Klawonn et al., 2015). Consequently, low-oxygen microzones within aggregates likely provide loci where the nitrogenase enzyme is protected from oxygen (Figure 1B). (2) *Metabolic energy*: Aggregates are characterized by elevated levels of labile organic carbon relative to the surrounding waters and rapidly become colonized by diverse bacteria. Enzymatic hydrolysis of the aggregate matrix allows ample carbon and nutrient supply and extensive microbial growth (Ploug and Grossart, 2000). This organic-rich microenvironment can, therefore, support the high energy requirements associated with diazotrophy by NCDs. (3) *Reactive nitrogen availability*: The high C:N ratio of aggregates (Figure 1C), and the consequent reduction in nitrogen availability due to microbial growth, may provide NCDs a competitive edge over other bacteria unable to fix N_2_. (4) *Trace metal and phosphorus availability:* Diazotrophy requires trace metals such as iron (Berman-Frank et al., 2001) and molybdenum (Marino et al., 2003). Since aggregates usually contain higher concentrations of trace metals than ambient water (Jackson and Burd, 1998; Engel et al., 2004), inhabiting diazotrophs may gain efficient access to these nutrients, in particular in the presence of increased microbial activity. Based on these observations, it may be surmised that aggregates can provide conditions that are beneficial for N_2_ fixation by NCDs.

## NCDs Associated With Aggregates: Towards a Conceptual Framework

Aggregates may provide favorable conditions for NCDs under various conditions in marine and freshwater environments. Yet, how these conceivably ephemeral conditions develop on aggregates and how NCDs exploit them is currently unclear. Based on the overall emerging picture outlined above, and recent experimental (Martínez-Pérez et al., 2018; Paerl et al., 2018) and modeling work (Chakraborty et al., 2021), we suggest a conceptual framework for N_2_ fixation by NCDs associated with aggregates (Figure 1D).

In the photic, well-oxidized zone, newly formed aggregates are sparsely colonized by microorganisms, thus limited respiration is expected. At that time, diffusion of oxygen from the surrounding water, and potential photosynthesis by associated phototrophs, will keep the aggregates well oxidized. If N_2_ fixation takes place, it is likely carried out mostly by associated cyanobacterial diazotrophs (Farnelid et al., 2018; Klawonn et al., 2019). It may be speculated that aggregate associated cyanobacterial diazotrophs can switch to mixotrophic metabolism to sustain N_2_ fixation as they sink to aphotic layers and photosynthesis is impaired (e.g., Rahav et al., 2013). Over time, aggregate-associated heterotrophic bacteria will proliferate, while preferentially exploiting labile nitrogen-rich organic compounds (Smith et al., 1992; Schneider et al., 2003), growing to cell concentrations commonly several orders of magnitude higher than in the surrounding water (Grossart and Simon, 1993; Turley and Mackie, 1994). This raises the C:N values of the aggregate over the Redfield ratio, gradually generating local nitrogen limitation (Figure 1C). At the same time extensive bacterial respiration exceeds the influx of oxygen diffusing from the surrounding water and causes formation of low oxygen microzones within the aggregate (Figure 1B). There is now a window of opportunity for N_2_ fixation by NCDs fueled by aerobic respiration. The low oxygen microzones may become anoxic if extensive bacterial respiration continues and exceeds the diffusive oxygen flux into the particle from the surrounding environment. NCDs may then switch to anaerobic respiration using nitrate or sulfate as alternative electron acceptors to meet their energetic requirements, as has been described for other aggregate-associated microbial processes (Wright et al., 2012; Bianchi et al., 2018) and recently modeled for NCDs (Chakraborty et al., 2021). The usual inhibition of N_2_ fixation by nitrate can be outweighed by enhanced diazotroph growth under low N:P ratio conditions (i.e., phosphate in excess; Knapp, 2012). However, it is unknown whether the high nitrate levels in deep waters may affect aggregate-associated NCD activity. It has been suggested that due to the high energetic costs associated with nitrate reduction, bacteria designed for diazotrophy may have few ecological reasons to use nitrate as a nitrogen source (Sprent and Sprent, 1990). In addition that high cell concentration near the surface of the aggregate may exhaust the supply of nitrate to the aggregate interior, supporting prevalence of sulfate over nitrate respiration within the aggregate (Chakraborty et al., 2021). Eventually, most of the labile carbon is consumed and heterotrophic respiration decreases. At that time, oxygen levels in the aggregate increase as oxygen consumption is exceeded by its diffusion from the surrounding water leading to significant reduction in N_2_ fixation rates by NCDs.

This conceptual framework for the interaction between NCDs and the dynamic environment on aggregates was recently modeled and yielded N_2_ fixation rates comparable to bulk rates measured in aphotic waters (Chakraborty et al., 2021), and agrees with field observations (Rahav et al., 2013, 2015; Benavides et al., 2016). Factors such as the level and type of substrate within the aggregate, the size of the aggregate, and its sinking speed may regulate the extent of aggregate associated N_2_ fixation both directly or indirectly, as they modulate the placement of the aggregate within the vertical gradients of nitrate, oxygen and carbon in the water column (Klawonn et al., 2015; Bianchi et al., 2018; Chakraborty et al., 2021).

## New Approaches and Methods

### How Much N_2_ Do NCDs Fix on Aggregates?

N_2_ fixation rates in aquatic environments are most commonly measured by ^15^N_2_ stable isotope labeling. Methodological challenges such as incomplete gas dissolution during incubations (Mohr et al., 2010) or contaminated gas stocks (Dabundo et al., 2014) causing under- or over-estimates of N_2_ fixation appear resolved (White et al., 2020). NCD-specific N_2_ fixation rates measurements have, however, remained elusive due to the coexistence of NCDs with cyanobacterial diazotrophs (Moisander et al., 2017). Approaches to distinguish the NCD N_2_ fixation signal from bulk rates have included dark incubations (Singh et al., 2021) and the addition of photosynthesis blocking agents (Rahav et al., 2016; Benavides et al., 2018b; Geisler et al., 2020). Unfortunately, these approaches cannot unambiguously measure NCD-specific N_2_ fixation rates since NCDs may be photoheterotrophic (Riemann et al., 2010). Moreover, blocking photosynthesis may not halt cyanobacterial N_2_ fixation at the expense of carbon storage, and alter the natural oxygen concentrations in incubation bottles (Table 1). Sample enrichment with ^15^N_2_ followed by nanoscale secondary ion mass spectrometry (nanoSIMS) yields cell-specific N_2_ fixation rates (Angel et al., 2018; Martínez-Pérez et al., 2018). The combination of nanoSIMS with phylogenetic or functional identity methods provides phylogenetic-specific N_2_ fixation rates (Musat et al., 2012), but hybridization preparations can dilute isotope signals impacting detectability when N_2_ fixation rates are low (Musat et al., 2014; Meyer et al., 2020; Table 1). Alternatives to circumvent this issue include correlation microscopy approaches and non-halogenated probes (gold-ISH; Kubota et al., 2014; Jiang et al., 2016; Table 1). In addition to the above, tagging the aggregate itself, while maintaining its structure during sample preparation for NanoSIMS or any other electron-based microscopy is highly challenging and calls for the development of dedicated sample preparation and imaging approaches.

**Table 1:** Proposed methods to study aggregate-associated NCDs.

Information sought	Method	Disadvantages/challenges	References
Bulk NCDs: N_2_ fixation rates	Dark incubations and/or photosynthesis inhibition, EA-IRMS	Photoheterotrophic NCDs downplayed Oxygen concentrations can change in closed incubations	Rahav et al., 2016; Benavides et al., 2018b; Singh et al., 2021
Aggregate-associated NCDs: N_2_ fixation rates	Sediment trap slurry or hand-picked aggregate ^15^N_2_ incubations, HISH-SIMS, correlation microscopy	Hybridization protocols cause isotope dilution impeding measurement of low rates Low throughput	Kubota et al., 2014; Dekas et al., 2016; Jiang et al., 2016; Loussert-Fonta et al., 2020
Aggregate-associated NCDs: phylogenetic and/or functional identity	CARD-FISH	Not optimal when phylogenetic diversity is high No active N_2_ fixation information	Biegala and Raimbault, 2008; Agawin et al., 2014
	Immunolabeling	No phylogenetic information	Geisler et al., 2019
	geneFISH	Combination of RNA-targeted oligonucleotide probes to infer cell identity with polynucleotide probes targeting gene fragments. Limited sensitivity	Moraru et al., 2010
Aggregate colonization architecture	Resin embedding, microtomy	Labor-intensive, compromised structure after dehydrating the sample, limited replicability and spatiotemporal extrapolation	Flintrop et al., 2018; Rogge et al., 2018
Spatial and temporal extrapolation	Laser *In-Situ* Scattering and Transmissometer (LISST), Underwater Video Profiler (UVP), holography, particulate optical backscattering	Aggregates containing diazotrophs not differentiated from others	Stemmann et al., 2012; Briggs et al., 2020; Walcutt et al., 2020

Note that a complete evaluation of the link between aggregates and diazotrophs using direct approaches will often also require complementary and indirect measurements.

### What is the Distribution and Spatial Organization of NCDs on and Within Aggregates?

NCDs may be localized on single aggregates using various tagging methods. Immunolabeling of the nitrogenase enzyme is a universal method to detect active nitrogenases (Geisler et al., 2019). The localization of diazotrophs on the particle could be achieved by tagging the aggregate matrix and immunolabeling the diazotrophs (Geisler et al., 2019). Moreover, NCDs can be differentiated from cyanobacteria by superimposing nitrogenase immunolabeling and phycoerythrin fluorescence images (Geisler et al., 2019). This approach does, however, not provide phylogenetic information. Yet, the biochemical heterogeneity and chemical gradients within aggregates (Ploug, 2001; Klawonn et al., 2015) likely regulate the distribution of phylogenetically and functionally distinct microbes (Wright et al., 2012). This implies that the colonizing architecture of NCDs on aggregates needs to be considered from a 3D perspective. This may be partially approached by laser scanning confocal microscopy (Geisler et al., 2019, 2020) and/or other approaches such as resin embedding followed by microtome slicing and 3D image reconstruction to investigate the internal aggregate structure (Flintrop et al., 2018; Rogge et al., 2018).

### Who Are the NCDs That Colonize Aggregates?

Barcoding, genomic and transcriptomic analyses have been applied on concentrated aggregate samples such as sediment trap material (Farnelid et al., 2018; Boeuf et al., 2019; Baumas et al., 2021). Such bulk approaches do, however, not allow visualizing the distribution of individual taxa at the single aggregate level. This would require specific methods such as rRNA oligonucleotide probes (catalyzed reporter deposition fluorescent *in situ* hybridization or CARD-FISH, e.g., (Thompson et al., 2012) and/or in combination with polynucleotide probes targeting specific gene fragments (geneFISH), which allows identifying individual phylogenetic groups expressing a gene of interest (Moraru et al., 2010). Recently, geneFISH was successfully used to quantify Gamma-A heterotrophic diazotrophs on marine aggregates (Harding, 2021).

### How Important Is Aggregate-Associated N_2_ Fixation by NCDs for Aquatic Nitrogen Cycling?

Traditional approaches to sample aggregate-associated microbes include hand-picking by SCUBA diving (Alldredge and Gotschalk, 1988) and size-fractionation (Mestre et al., 2017). Given the heterogeneous distribution of aggregates in water columns, small volume sampling devices such as Niskin bottles underestimate aggregate abundance causing a bias towards free-living microbes and dissolved materials (Planquette and Sherrell, 2012; Puigcorbé et al., 2020). A plethora of devices that integrate larger water volumes such as *in situ* pumps, marine snow catchers and sediment traps exist today (McDonnell et al., 2015). While these provide a better representation of aggregate abundances and distributions in the water column, the downstream analyses proposed above to yield NCD-specific metabolic and phylogenetic information are mostly low throughput (Table 1). Extrapolating low throughput discrete measurements to large spatial and temporal scales would require, on top of a sufficiently representative sampling, knowledge on aggregate size spectra and spatiotemporal distribution (Boyd et al., 2019; Giering et al., 2020). The advent of automated aggregate counting and imaging methods (Stemmann et al., 2012; Giering et al., 2020; Karlusich et al., 2021) will likely improve the accuracy of spatiotemporal scale extrapolations in the future.

## Epilog: Heterotrophic Diazotrophs Associated With Aggregates

We argue that aggregates act as dynamic loci suitable for N_2_ fixation by NCDs in aquatic ecosystems. Molecular analyses and microscopical identification have shown that material collected in large size fractions and sediment trap material harbor clusters of NCDs. However, the phylogeny, the specific N_2_ fixation rates of NCDs on aggregates and their contribution to nitrogen cycling remain largely unquantified. It is, therefore, important to develop dedicated methods and approaches capable of isolating NCD-specific N_2_ fixation rates and to identify their phylogeny. Our recommendation to the scientific community is to (1) develop cell-specific staining methods combined with ^15^N_2_ labeling, (2) consider the 3D architecture of single aggregates, and (3) account for their heterogeneous spatiotemporal distribution in aquatic ecosystems. Advances in automated particle characterization and counting should increase the throughput of these methods in the future. These recommendations will inspire future research to unveil the ecology and quantitative relevance of aggregate-associated NCDs in marine as well as freshwater environments.

## Author Contributions

LR, ER, MB, and EB-Z wrote the manuscript. UP, H-PG, DB, IK, and ME commented on the final version of the manuscript. All authors contributed to the article and approved the submitted version.

## Funding

MB and LR were supported by the BNP Paribas Foundation for Climate and Diversity grant “NOTION.” LR was supported by the Danish Council for Independent Research (6108-00013B). EB-Z was supported by the Israeli Science Foundation (grant number 944\21). H-PG was supported by the German Science Foundation (GR1540/28-1 and 37-1).

## Conflict of Interest

The authors declare that the research was conducted in the absence of any commercial or financial relationships that could be construed as a potential conflict of interest.

## Publisher’s Note

All claims expressed in this article are solely those of the authors and do not necessarily represent those of their affiliated organizations, or those of the publisher, the editors and the reviewers. Any product that may be evaluated in this article, or claim that may be made by its manufacturer, is not guaranteed or endorsed by the publisher.
